# Microstrain Analysis of Selective Pressure Techniques for Mandibular Complete Denture Impression: An In Vivo Study

**DOI:** 10.7759/cureus.23673

**Published:** 2022-03-31

**Authors:** Ashish Dadarwal, Jyoti Paliwal, Vineet Sharma, Kamal K Meena, Ramjee L Raigar, Mohini Gaziwala

**Affiliations:** 1 Prosthodontics, Rajasthan University of Health Sciences (RUHS) College of Dental Sciences, Jaipur, IND

**Keywords:** relief, spacer, complete denture, microstrain analysis, selective pressure

## Abstract

Aim

The present in vivo study was undertaken for microstrain analysis of the selective pressure impression technique using strain gauges in mandibular complete denture impression.

Methodology

Ten completely edentulous patients undergoing complete denture treatment were selected for the study. For each patient, mandibular casts were made, and two custom trays were fabricated on the mandibular cast. These mandibular trays were divided into two groups: those with mandibular impressions made in the custom tray with the use of a spacer (MST) and those without a spacer (MWS). For each patient, a primary impression was made by using an impression compound. After that, the primary cast was obtained, and the custom tray was fabricated by using auto polymerizing resin. Then strain gauges were attached to the particular areas of the tray. The customized tray with zinc oxide eugenol impression material was seated in the patient's mouth for the final impression. The strain produced during impression making at different areas was recorded by a multi-channel (six) strain amplifier and data logger (8-channel digital strain indicator NIC, Jaipur, India). The data obtained were subjected to statistical analysis using an independent t-test (for quantitative data within two groups). The level of significance was set at p=0.05 (p<0.05-significant, p<0.01-highly significant, p<0.0001-very highly significant).

Results

The comparison of the percentage of microstrain produced at the relief area with and without spacer tray design showed a statistically significant (p=0.001) result with a greater number of percentages of microstrain among those without spacers (94.19) than with spacer (72.09) tray design.

Conclusion

The use of a tray with relief for selective pressure impression of an edentulous mandible resulted in a desirable pressure distribution at the alveolar crests and buccal shelves.

## Introduction

An impression is a record of the negative form of the tissues of the oral cavity that make up the basal seat of the denture. All possible methods should be undertaken to ensure good tissue health by minimizing potential traumatic effects on a complete denture wearer [1]. Different authors have proposed various theories to achieve an optimal impression in terms of pressure on the underlying tissue. The most common are the mucostatic, mucocompressive, and selective pressure techniques [2-5].

The mucostatic technique, also known as pressureless impression, is the negative duplication of oral tissues at rest with no soft tissue displacement [6-8]. The mucostatic theory is based on the belief that the oral tissues of the denture bearing area behave as a confined fluid following Pascal's law of hydrostatics. These laws state that pressure exerted on a confined liquid will be distributed evenly throughout the fluid. Similarly, the tissue fluid under the denture behaves like a confined liquid wherein the force on the denture is counteracted by the buoyancy of the tissue fluid flowing from one area to another. This technique has advantages like excellent retention due to closed mucosal adaption, less trauma to the tissue, and a decreased chance of residual ridge resorption, but they also have the disadvantage of poor stability during occlusal load [9].

The mucocompressive technique, on the other hand, is based on the principle of making an impression of tissues under controlled pressure. They would withstand functional forces if the tissue was recorded in a compressed state. Some proponents of this technique claim that the pressure exerted on the tissues will restrict blood flow, creating bone resorption and tissue irritation, as well as causing a rebound of the prosthesis while it is not functioning. This often leads to good initial retention, but ultimately resorption and a loose denture [5].

The selective pressure impression technique is based on the selective pressure theory advocated by Carl Boucher [10] combines aspects of both techniques. This philosophy of impression is based on the histological understanding of supporting tissues. Areas such as posterolateral slopes of the hard palate or buccal shelf in the mandible, which are anatomically favorable to withstand pressure, are loaded. These areas are supported by the dense cortical bone and covered by a variable-thickness mucous membrane. The denture supporting area is divided into primary, secondary, and non-stress bearing areas. The rugae, midline raphe, crest of the mandibular alveolar ridge, and areas of movable tissue are relieved because they do not provide the same favorable anatomic and histological quality for withstanding functional load. After all, these areas are covered by a thin layer of the mucous membrane [10]. The selective pressure impression technique is based on the selective pressure theory advocated by Carl Boucher combines aspects of both techniques. This philosophy of impression is based on the histological understanding of the supporting tissues. Loaded areas include the posterolateral slopes of the hard palate and the buccal shelf in the mandible, which are anatomically favorable to withstand pressure. These areas are supported by the dense cortical bone and covered by a variable-thickness mucous membrane. The denture-supporting area is divided into primary, secondary, and non-stress-bearing areas. The rugae, midline raphe, crest of the mandibular alveolar ridge, and areas of movable tissue are relieved because they do not provide the same favorable anatomical and histological quality for withstanding functional load. After all, these areas are covered by a thin layer of the mucous membrane [10].

Although various methods for making selective pressure impressions for edentulous patients have been reported, the unfortunate fact remains that nearly everything that has been written about is based on empirical knowledge. Almost no research work has been published, either in the laboratory or in the clinic [11]. The success of these techniques varies according to the clinician's experience with different impression materials and tray spacer designs. Howell and Manly [12] used an electronic strain gauge for measuring oral forces. With the help of an unbounded strain gauge, Brudevold [13] measured vertical force in the mouth of a denture wearer during normal, comfortable chewing with the help of an oscillograph. According to Frank [14], the impression pressure could be controlled by tray design and material choice. Using a mandibular simulated analog, Al-Ahmad [15] measured pressure during edentulous impression making at various locations. Reddy et al. [16] did an in vivo study using a bonded strain gauge of 120 ohms that gave a digital reading and concluded that the relief space of modeling wax reduces pressure on the residual ridge.

The pressure developed at the stress-bearing areas (buccal shelf area) and the relief area (crest of the residual alveolar ridge) of the mandible during impression making with two custom trays with and without relief spacer with zinc oxide eugenol impression paste were evaluated and compared using a strain gauge as suggested by selective pressure theory.

## Materials and methods

The study was carried out in the Department of Prosthodontics of the RUHS College of Dental Sciences in Jaipur, India. A total of 10 completely edentulous patients undergoing complete denture treatment were selected for the study after clearance from the Institutional Ethical Committee of the RUHS College of Dental Sciences. Completely edentulous patients with well-formed residual alveolar ridges without any tissue and bony undercut and with adequate interarch space, patients after six months of extraction, and patients who were able to follow the instructions were included. The study excluded patients with atrophied ridges, mucosal soreness and ulceration, bone spicules, severe tissue, bony undercut, neuromuscular disorder, and indifferent critical patients. The participant data was formulated and used for research purposes only. All procedures were thoroughly explained before participation.

**Figure 1:**
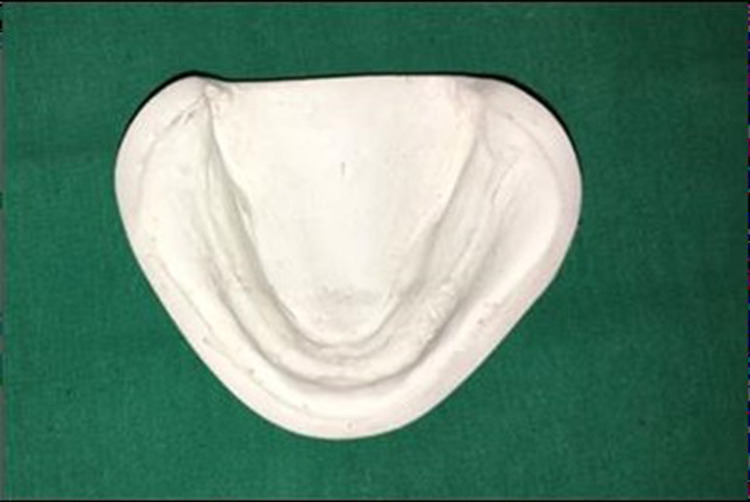
Mandibular primary cast without spacer

**Figure 2:**
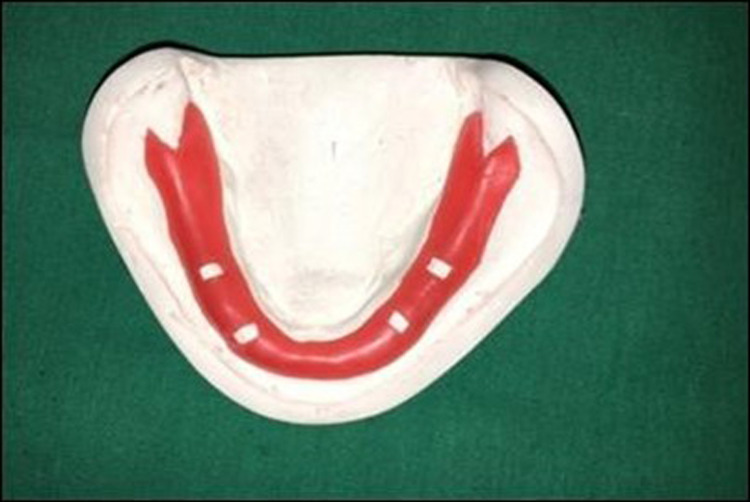
Mandibular primary cast with spacer

A model of an edentulous mandibular ridge was prepared using the mucocompressive impression technique with impression compound type I (Y-Dent; MDM Corp., Laguna Beach, California). The impression was then poured with type II dental stone (Kalstone; Kalabhai Karson Pvt. Ltd, Mumbai, India). The stone was allowed to set, and then the mandibular cast was removed and finished (Figure 1). For each patient, a mandibular cast was made and two custom trays were fabricated on the mandibular cast. These mandibular trays were divided into two groups: those with mandibular impressions made in the custom tray with the use of a spacer (MST) and those without a spacer (MWS). The outline of the border of the tray was marked 2-3 mm short of reflection with the pencil. Relief was given at the crest of the ridge area by adapting spacer wax of 1 mm thickness. Four tissue stops (2 mm in size) were placed in the molar and cuspid regions (Figure 2). A separating medium was applied with the help of a brush in a uniform thickness over the primary cast before the fabrication of the custom tray. When the dough reached the stage of rolling out, it was rolled out with the tray former to a uniform thickness of 2 mm. A full sheet of the material is then centered over each preliminary cast and gently adapted. Material that extended beyond the edge of the preliminary cast was removed with a sharp scalpel, and the material was re-adapted to the preliminary cast. A single handle was positioned in the midline of the lower tray.

**Figure 3:**
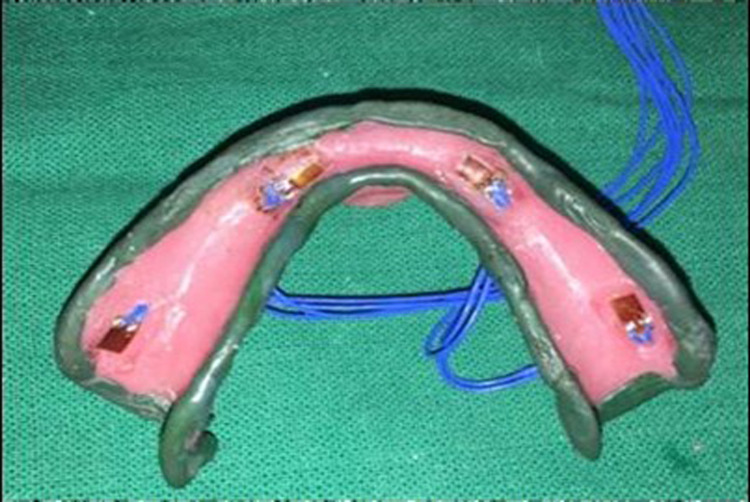
Border molding without spacer application

**Figure 4:**
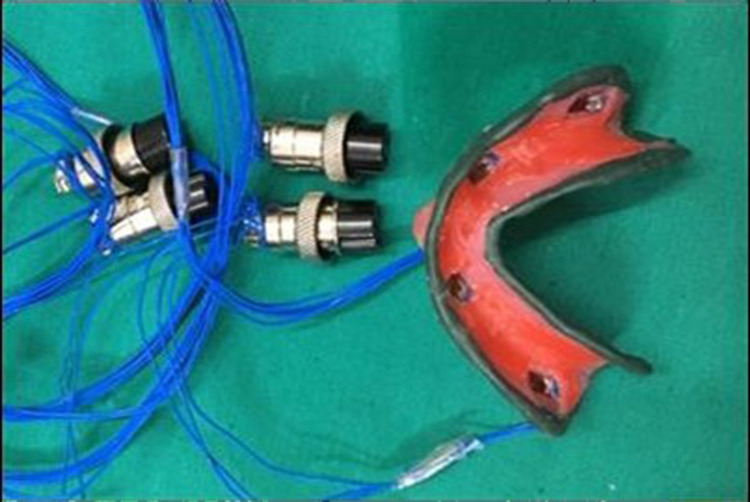
Border molding with spacer application

One hundred twenty ohm strain gauges (Strain gauge, NIC, Jaipur, India) were applied to the fabricated custom tray with the help of a pasting kit (Strain gauge pasting kit, NIC, Jaipur, India) at the appropriate locations (right buccal shelf region, right canine region, left canine region, left buccal shelf region) and numbered 1, 2, 3, and 4 respectively. The 600V polyvinyl chloride (PVC) insulated standard electric wire was soldered with an M line solder and connected to a multi-channel strain amplifier and data logger (8-channel digital strain indicator NIC, Jaipur, India) with PC-based real-item display and analysis software (Data acquisition systems, NIC, Jaipur, India). Functional border molding was done using a low-fusing impression compound (DPI Pinnacle tracing sticks; Dental Products of India, Mumbai, India) (Figure 3,4).

**Figure 5:**
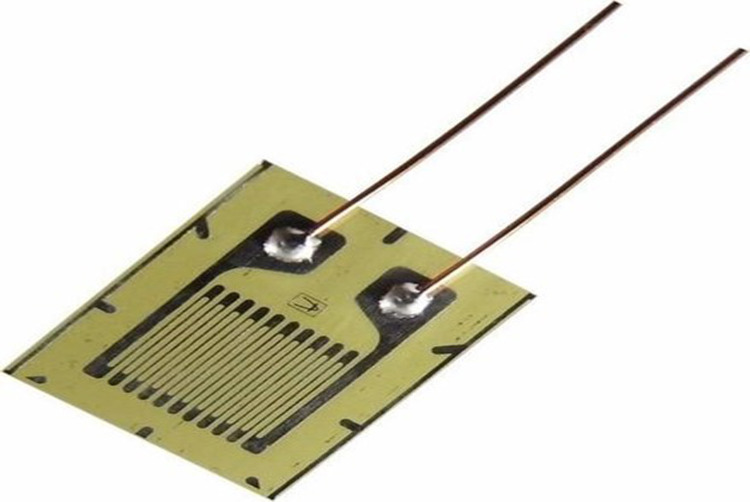
Strain gauge

**Figure 6:**
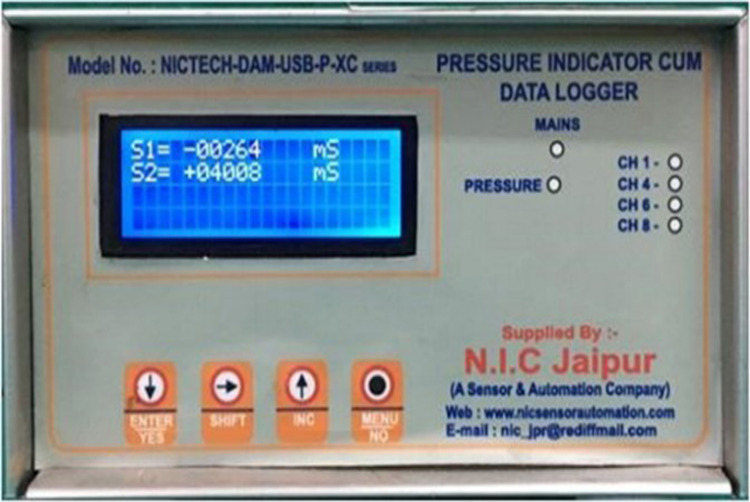
Digitizer cum data logger displaying microstrain values

**Figure 7:**
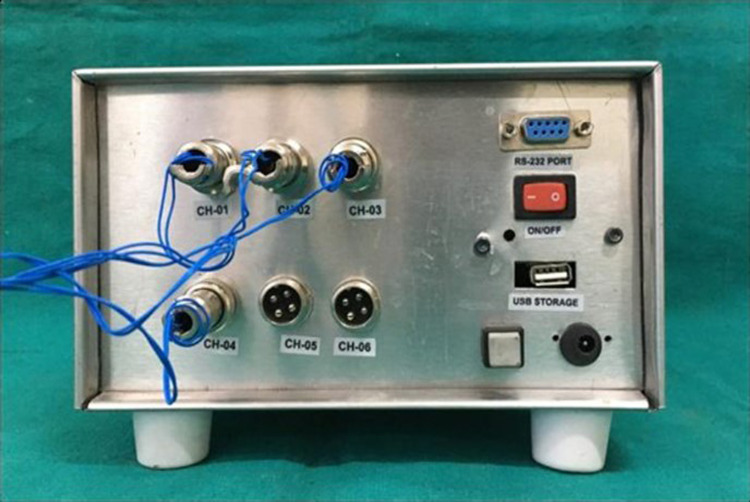
Digital data logger showing four channels connected with strain gauges

**Figure 8:**
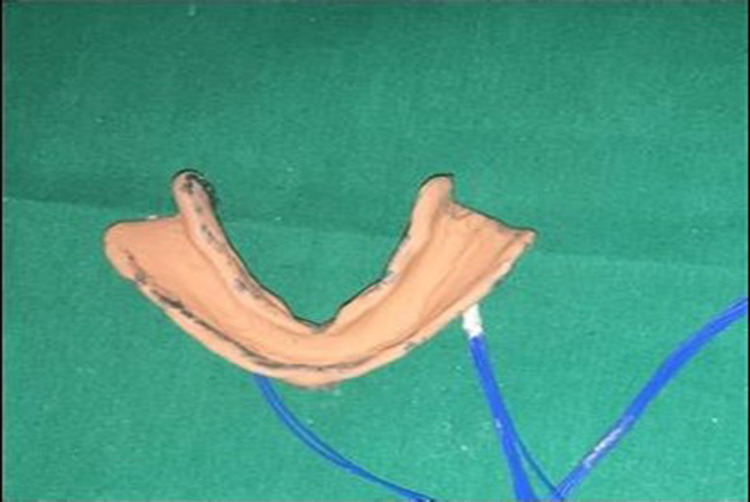
Final impression without spacer

**Figure 9:**
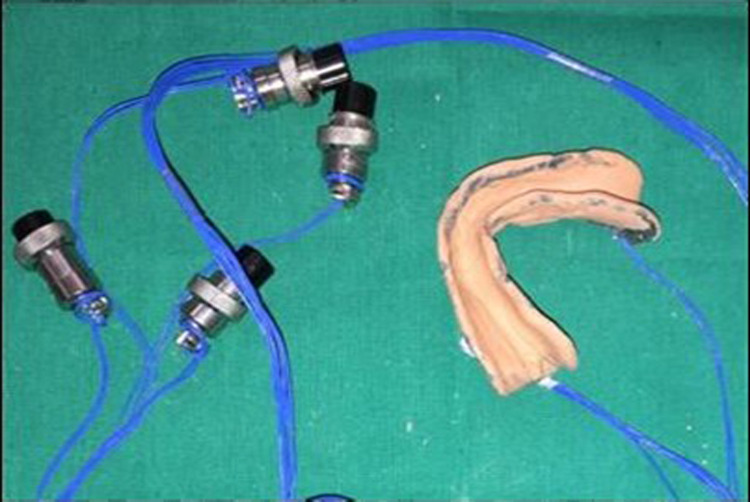
Final impression with spacer

After the completion of border molding, the custom tray with microwire extensions was attached to the data logger (six-channel with PC based real-time display and analysis software) via four connectors (Figure 5-7), and the final impression was made with zinc oxide eugenol impression material, and the tray was held in place with two-finger supports, i.e., index and middle finger in molar areas bilaterally. A total of 20 impressions were made (10 impressions with spacer and 10 impressions without spacer) (Figure 8,9). The data logger had a display screen that displayed the readings of pressure applied during impression making in terms of microstrain. Readings were noted at the time of the final set of the impression paste.

Statistical analysis

The data was coded and entered into an excel spreadsheet (Microsoft, Redmond, Washington). Analysis was done using SPSS version 20 (IBM Inc., Armonk, New York). The variables were assessed for normality using the Kolmogorov Smirnov test. Descriptive statistics include the computation of means and standard deviations. For quantitative data between two groups, the independent t-test was used. The level of significance was set at p=0.05. (p<0.05 - significant, p<0.01 - highly significant, p<0.0001 - very highly significant).

## Results

The microstrain was observed for all the subjects during impression making using custom trays designed with a spacer (MST) and without a spacer (MWS) (Table 1, 2). Comparison of microstrain in relief areas with and without spacer tray design showed a statistically significant (p=0.004) result with more mean value in MWS (897.07µE) than MST (597.36µE), and comparison of microstrain in non-relief areas (stress-bearing areas) with and without spacer tray design showed no significant result (Table 3).

**Table 1:** Study subject observation readings in microstrain (µE) for relief areas and non-relief areas with two tray designs (MST and MWS) MST - custom trays designed with a spacer; MWS - custom trays designed without a spacer

Subjects	Relief area reading in (µE)		Stress bearing or non-relief area reading in (µE)	
	MST	MWS	MST	MWS
1	423.5	736.75	613.20	850.32
2	635.01	989.5	865.3	932.7
3	750.21	1210.5	1080.31	1335
4	315.8	636.6	513.1	720.5
5	752.1	815.9	935.4	833.2
6	386.1	708.1	582.4	788.4
7	473.3	786.7	659.2	893.7
8	830.4	1290.7	1160.9	1405.3
9	782.2	845.4	961.7	865.3
10	625.04	950.6	820.4	905.6

**Table 2:** Study subject observation readings in average percentage for two tray designs with and without a spacer

Subject	With spacer % of strain at relief area compared to maximum strain 100% (stress-bearing)	Without spacer % of strain at relief area compared to maximum strain 100 % (stress-bearing)	% difference of strain at relief area with & without spacer
1	69.06	86.64	17.58
2	73.38	106.08	32.7
3	69.44	90.67	21.23
4	61.54	88.35	26.81
5	80.40	97.92	17.52
6	66.29	89.81	23.52
7	71.79	88.02	16.23
8	71.53	91.84	20.31
9	81.33	97.70	16.34
10	76.18	104.96	28.78

**Table 3:** Comparison of microstrain (A) Comparison of microstrain in relief areas with and without spacer tray design. (B) Comparison of microstrain in non-relief areas (stress-bearing area) with and without spacer tray design. (C) Comparison of microstrain in the tray with spacer design between relief areas and non-relief areas

Table	Group	N	Mean ± SD	P-value
A	With spacer	10	597.36 µE ± 184.83 µE	0.004
	Without spacer	10	897.07 µE ± 214.8 µE	
B	With spacer	10	819.19 µE ± 220.77 µE	0.2
	Without spacer	10	953.03 µE ± 228.62 µE	
C	Relief area	10	597.36 µE ± 184.83 µE	0.02
	Non-relief area	10	819.19 µE ± 220.77 µE	

Comparison of microstrain in the tray with spacer design between relief areas and non-relief areas showed a statistically significant (p=0.02) result with more mean value in the non-relief area (819.19µE) than in the relief area (597.36µE) and comparison of microstrain in the tray without spacer design between relief areas and non-relief areas showed no statistically significant (p=0.58) result (Table 3,4).

**Table 4:** Comparison of microstrain (A) Comparison of microstrain in tray design without a spacer between relief areas and non-relief areas. (B) Comparison of percentage of microstrain produce at relief area with and without spacer tray design

Table	Group	N	Mean ± SD	P-value
A	Relief area	10	897.07 µE ± 214.8 µE	0.58
	Non-relief area	10	953.02 µE ± 228.62 µE	
B	With spacer	10	72.09 µE ± 6.09 µE	0.001
	Without spacer	10	94.19 µE ± 7.07 µE	

The comparison of the percentage of microstrain produced at the relief area with and without spacer tray design showed a statistically significant (p=0.001) result with a greater number of percentages of microstrain among those without spacers (94.19) than with spacer (72.09) tray design (Table 4).

## Discussion

In edentulous patients, bone resorption progresses much faster in the mandible than in the maxilla, and the ridges of the mandible undergo a variety of changes [17-19]. Inouse et al. [20] reported that bone density is lower in edentulous mandible alveolar crests when ridges are higher. Dentures should be designed to minimize masticatory pressure on mandibular alveolar crests to slow the progression of bone resorption in the edentulous mandible. As a result, when making an impression, avoid applying pressure to the alveolar crests. Because of masticatory pressure considerations, the buccal shelves have been used as the primary supporting areas in complete mandibular dentures [21,22]. The amount and distribution of pressure beneath prosthodontic impressions have been the subject of academic debate and research for a very long time. Of the various impression techniques, selective pressure impression techniques focus on controlling pressure exerted on the oral mucosa to provide superior results. If the amount and location of pressure produced during impression making are controlled, the prognosis of the complete denture will be better. Certain areas of the edentulous ridge should be stressed while others should be relieved, according to proponents of selective pressure techniques [23].

The present study was done to evaluate the microstrain produced at the buccal shelf area of the ridge and crest of the residual alveolar ridge while making an impression with two tray designs, i.e., with and without a spacer. Strain gauges were applied to the fabricated custom tray. For more than 60 years, strain gauges have been used intraorally for measuring pressure. Strain gauge has been used for microstrain analysis of occlusal forces on implant prostheses and even strain in dental braces. As early as 1951, Brudevold [13] used to insert small strain gauges under individual artificial teeth to form bite elements that are cemented into the denture base in proper occlusion. These strain gauges were unbonded, and an oscillograph was used for the output mechanism, but digital reading was not possible with these strain gauges. To evaluate the force during clenching, Stromberg [24] used a strain gauge instrument with a gold spring attached to a movable acrylic resin window constructed in the lateral part of the maxillary denture base in 1955. These gauges were connected to a brush strain analyzer, which provides a source of power for the bridge, as well as an amplifier. Through the use of a brush pen recorder, a written record of the applied forces can be obtained. Digital reading was not possible with these strain gauges, so the procedure was time-consuming. In an in-vitro study in 1969, Frank [14] used an unbonded strain gauge as a pressure transducer and an oscillograph as the output mechanism in an in-vitro study to analyze the pressures produced during maxillary edentulous impression procedures. The apparatus used in the frank study was very complicated, time-consuming, and could not be used intra-orally. Digital reading was not possible with these strain gauges.

This study's apparatus was similar to that used by Reddy et al. [16] in 2013. In both studies, a bonded strain gauge of 120 ohms was used that gives digital readings. In our study, strain gauges were attached directly to the inner side of the custom tray, which is good for precision measurement. As the custom tray was deformed, the strain gauge foil was deformed, causing its electrical resistance to change. The data logger converts mechanical strain into a digital reading. It was found that the use of spacers resulted in a significant reduction in the strain values in the mandibular alveolar crest area (relief area) in comparison with the buccal shelf area, i.e., 22.03 percent on average with a p-value of 0.001 (highly significant). Mean comparison microstrain produced in the relief area between with and without spacer tray design. A statistically significant (p=0.004) result was found with more mean value without spacer (897.07µE) than with spacer (597.36µE). Concerning the mean comparison of microstrain produced in the non-relief area between with and without spacer tray design, statistically, no significant (p=0.2) result was found with more mean value without spacer (953.03) than with spacer (819.19). Mean comparison of microstrain produced in with spacer tray design between relief and non-relief area. This study's apparatus was similar to that used by Reddy et al. [16] in 2013. In both studies, a bonded strain gauge of 120 ohms was used that gives digital readings. In our study, strain gauges were attached directly to the inner side of the custom tray, which is good for precision measurement. As the custom tray was deformed, the strain gauge foil was deformed, causing its electrical resistance to change. The data logger converts mechanical strain into a digital reading. It was found that the use of spacers resulted in a significant reduction in the strain values in the mandibular alveolar crest area (relief area) in comparison with the buccal shelf area, i.e., 22.03 percent on average with a p-value of 0.001 (highly significant). Mean comparison microstrain produced in the relief area between with and without spacer tray design. A statistically significant (p=0.004) result was found with more mean value without spacer (897.07) than with spacer (597.36). Concerning the mean comparison of microstrain produced in the non-relief area between with and without spacer tray design, statistically, no significant (p=0.2) result was found with more mean value without spacer (953.03) than with spacer (819.19). Mean comparison of microstrain produced in with spacer tray design between relief and non-relief area. Statistically significant (p=0.02) results were found with more mean value in the non-relief area (819.19) than in the relief area (597.36). Mean comparison of microstrain produced in the tray design without a spacer between relief and non-relief area. Statistically, no significant (p=0.58) result was found with more mean value in the non-relief area (953.03) than in the relief area (897.07).

Sayumi et al. [25] found that impression pressure on buccal shelves in trays with 1.4 mm of relief and escape holes at the alveolar crests was significantly higher than that on the alveolar crests in all impression materials. He also claimed that increasing relief thickness expands the space through which impression material flows near the mandibular alveolar crests, thereby reducing impression pressure [25]. Al-Ahmad et al. [15] did an in vitro study to measure the pressure exerted under a simulated mandibular edentulous impression at different locations using commonly used impression material and four impression tray configurations. He found that there was a significant difference in pressure production among the four different trays. A tray with no relief and no holes produced the highest pressure; this pressure was significantly higher than that produced by trays that had no relief with holes, relief with no holes, and relief with holes. No significant difference in pressure was found between the trays that had no relief with holes and the trays that had relief with and without holes [15]. Our results were also similar to the above-mentioned studies and showed impression pressure on alveolar crests was lower for trays with relief spacers than for those without. The use of spacers was found to selectively increase pressure at the buccal shelf area (22.3 percent on average) in comparison to the mandibular alveolar crest area (relief area).

Sayumi et al. [25] and Al-Ahmad et al. [15] found that impression pressure was higher at the median alveolar crest than at the buccal shelf area in the tray without a spacer, but in our study, the result was found with more microstrain value in the buccal shelf area (953.03) than in the alveolar crest area (897.07). There may be several reasons why there is a difference in results between this study and that of Al-Ahmad et al. and Sayumi et al. The main difference between these studies is that our study was in vivo, and the pressure was applied manually with fingers at the first molar areas. The mentioned studies were in vitro, and pressure was applied with a machine. Impression pressure in edentulous jaws has been studied in several studies. The impression pressure in the edentulous maxilla was studied by Frank et al., who found that it varies depending on the tray design [14]. Inoue et al. investigated the effect of relief thickness on impression pressure using a clinical simulation model of an edentulous maxilla and discovered that relief equalized impression pressure in the tray [20]. When making an impression of an edentulous maxilla, Komiyama et al. [26] suggested using an escape hole of 1.0 mm or larger, or a spacer the thickness of a sheet of base plate wax, to selectively reduce palatal impression pressure. Reddy et al. [16] did an in vivo study and reported that a relief space of one sheet thickness of modeling wax effectively reduced pressure exerted on the maxillary residual alveolar ridge while making edentulous impressions.

A limitation of the present clinical study is the small sample size. By measuring a larger sample size, factors like gender, age, and the number of years of edentulousness might have been identified. Furthermore, additional research can be carried out by modifying variables such as the escape holes for relief, spacer thickness, different spacer designs, and impression materials.

## Conclusions

These findings suggested that clinicians can select pressure and non-pressure areas while making impressions for complete dentures. The use of a tray with relief for selective pressure impression of edentulous mandible results in pressure distribution in which impression pressure at the alveolar crests is reduced while the buccal shelves carry masticatory force. The present findings are useful for impression techniques that account for the prevention of bone resorption in edentulous mandibular ridges.
